# *ABCC5*, a Gene That Influences the Anterior Chamber Depth, Is Associated with Primary Angle Closure Glaucoma

**DOI:** 10.1371/journal.pgen.1004089

**Published:** 2014-03-06

**Authors:** Monisha E. Nongpiur, Chiea Chuen Khor, Hongyan Jia, Belinda K. Cornes, Li-Jia Chen, Chunyan Qiao, K. Saidas Nair, Ching-Yu Cheng, Liang Xu, Ronnie George, Do Tan, Khaled Abu-Amero, Shamira A. Perera, Mineo Ozaki, Takanori Mizoguchi, Yasuo Kurimoto, Sancy Low, Liza-Sharmini A. Tajudin, Ching-Lin Ho, Clement C. Y. Tham, Ileana Soto, Paul T. K. Chew, Hon-Tym Wong, Balekudaru Shantha, Masako Kuroda, Essam A. Osman, Guangxian Tang, Sujie Fan, Hailin Meng, Hua Wang, Bo Feng, Victor H. K. Yong, Serena M. L. Ting, Yang Li, Ya-Xing Wang, Zheng Li, Raghavan Lavanya, Ren-Yi Wu, Ying-Feng Zheng, Daniel H. Su, Seng-Chee Loon, R. Rand Allingham, Michael A. Hauser, Nagaswamy Soumittra, Vedam L. Ramprasad, Naushin Waseem, Azhany Yaakub, Kee-Seng Chia, Govindasamy Kumaramanickavel, Tina T. Wong, Alicia C. How, Tran Nguyen Bich Chau, Cameron P. Simmons, Jin-Xin Bei, Yi-Xin Zeng, Shomi S. Bhattacharya, Mingzhi Zhang, Donald T. Tan, Yik-Ying Teo, Saleh A. Al-Obeidan, Do Nhu Hon, E-Shyong Tai, Seang-Mei Saw, Paul J. Foster, Lingam Vijaya, Jost B. Jonas, Tien-Yin Wong, Simon W. M. John, Chi-Pui Pang, Eranga N. Vithana, Ningli Wang, Tin Aung

**Affiliations:** 1 Singapore Eye Research Institute and Singapore National Eye Centre, Singapore; 2 Infectious Diseases, Genome Institute of Singapore, Singapore; 3 Human Genetics, Genome Institute of Singapore, Singapore; 4 Department of Paediatrics, National University Health System & National University of Singapore, Singapore; 5 Saw Swee Hock School of Public Health, National University of Singapore, Singapore; 6 Beijing Tongren Eye Center, Beijing Tongren Hospital, Capital Medical University, Beijing Ophthalmology & Visual Sciences Key Lab, Beijing, China; 7 Department of Ophthalmology & Visual Sciences, the Chinese University of Hong Kong, Hong Kong, China; 8 Howard Hughes Medical Institute, The Jackson Laboratory, Bar Harbor, Maine, United States of America; 9 Department of Ophthalmology, National University Health System & National University of Singapore, Singapore; 10 Beijing Institute of Ophthalmology, Beijing Tongren Hospital, Capital University of Medical Science, Beijing, China; 11 Vision Research Foundation, Sankara Nethralaya, Chennai, India; 12 Vietnam National Institute of Ophthalmology, Hanoi, Vietnam; 13 Department of Ophthalmology, College of Medicine, King Saud University, Riyadh, Saudi Arabia; 14 Ozaki Eye Hospital, Hyuga, Japan; 15 Mizoguchi Eye Clinic, Sasebo, Japan; 16 Kobe Gen Hospital, Kobe, Japan; 17 NIHR Biomedical Research Centre for Ophthalmology at Moorfields Eye Hospital and UCL Institute of Ophthalmology, London, United Kingdom; 18 Department of Ophthalmology, School of Medical Sciences, Universiti Sains Malaysia, Kota Bharu, Kelantan, Malaysia; 19 Department of Ophthalmology, Tan Tock Seng Hospital, Singapore; 20 Xingtai Eye Hospital, Xingtai, China; 21 Handan Eye Hospital, Handan, China; 22 Anyang Eye Hospital, Anyang, China; 23 Duke University Medical Center, Durham, North Carolina, United States of America; 24 Oxford University Clinical Research Unit, Ho Chi Minh City, Vietnam; 25 Centre for Tropical Medicine, Nuffield Department of Clinical Medicine, Oxford University, Oxford, United Kingdom; 26 State Key Laboratory of Oncology in Southern China, Guangzhou, China; 27 Department of Experimental Research, Sun Yat-Sen University Cancer Centre, Guangzhou, China; 28 Peking Union Medical College, Chinese Academy of Medical Science, Beijing, China; 29 Shantou University/Chinese University of Hong Kong Joint Shantou International Eye Center, Shantou, China; 30 Department of Medicine, National University Health System & National University of Singapore, Singapore; 31 Department of Ophthalmology, Medical Faculty Mannheim of the Ruprecht-Karls-University Heidelberg, Germany; 32 Beijing Institute of Ophthalmology, Beijing Tongren Hospital, Capital Medical University, Beijing, China; Harvard University, United States of America

## Abstract

Anterior chamber depth (ACD) is a key anatomical risk factor for primary angle closure glaucoma (PACG). We conducted a genome-wide association study (GWAS) on ACD to discover novel genes for PACG on a total of 5,308 population-based individuals of Asian descent. Genome-wide significant association was observed at a sequence variant within *ABCC5* (rs1401999; per-allele effect size = −0.045 mm, *P* = 8.17×10^−9^). This locus was associated with an increase in risk of PACG in a separate case-control study of 4,276 PACG cases and 18,801 controls (per-allele OR = 1.13 [95% CI: 1.06–1.22], *P* = 0.00046). The association was strengthened when a sub-group of controls with open angles were included in the analysis (per-allele OR = 1.30, *P* = 7.45×10^−9^; 3,458 cases vs. 3,831 controls). Our findings suggest that the increase in PACG risk could in part be mediated by genetic sequence variants influencing anterior chamber dimensions.

## Author Summary

The anterior chamber is the space within the eye which is bound by the cornea, and the anterior surfaces of the iris and lens. Anterior chamber depth (ACD) is the distance measured along the eye's optical axis, from the cornea to the lens surface. ACD is an important risk factor for primary angle closure glaucoma (PACG), a major cause of irreversible blindness worldwide, and in particular, individuals of Asian ethnicity. In order to identify the genes that underlie PACG susceptibility, we conducted a two-staged study. We first conducted a large scale genetic study on a total of 5,308 population-based individuals of Asian descent to identify the genetic variants that influence ACD. This was followed by testing for associations between the identified genetic variant and PACG in another independent collection of 4,276 PACG cases and 18,801 controls. We found that a genetic variant within *ABCC5* was associated with an increased risk of having PACG. Our findings suggest that the increase in PACG risk could in part be mediated by genetic sequence variants that influence the anterior chamber dimensions of the eye.

## Introduction

Primary angle closure glaucoma (PACG) remains a major cause of irreversible blindness, particularly in Asian countries such as China [1], Mongolia [2], Singapore [3], and India [4] with up to 80% of the estimated 15 million people afflicted with PACG resident in Asia [5]. We recently conducted a genome-wide association study (GWAS) on PACG with 3,771 PACG cases and 18,551 controls, and identified 3 strongly associated genetic variants: rs11024102 in *PLEKHA7*, rs3753841 in *COL11A1* and rs1015213 located between *PCMTD1* and *ST18* on Chromosome 8q [6]. As these 3 sequence variants only explained <2 percent of PACG risk, we looked into other methodologies besides the GWAS based approaches to identify more genes that underlie PACG susceptibility. The clinical heterogeneity of PACG suggests that disease-related endophenotypes/quantitative traits may help elucidate true disease genes. Quantitative phenotypes allow individuals to be viewed along the continuum of risk, and may provide additional information which could complement dichotomous measures of affection status [7], [8]. Such an approach has been used in the study of genetic variants controlling lipid traits and susceptibility to coronary artery disease [7], [8].

Smaller anterior segment dimensions are a hallmark of PACG, with shallower anterior chamber depth (ACD), the cardinal feature associated with increased susceptibility to PACG [9], [10]. Eyes with an ACD of less than 2.80 mm were more inclined to have angle closure when compared to eyes with an ACD of at least 3 mm (odds ratio (OR), 42.5; 95% confidence interval (CI), 27.4–66.2) [11]. There is also a greater likelihood of developing glaucomatous optic neuropathy in persons with the shallowest anterior chambers [12].

ACD, an easily and precisely quantified measure by ocular imaging techniques, is a normally distributed quantitative trait within the general population. It displays high heritability with a coefficient as high as 0.90 [13], [14], and can be considered an endophenotype for PACG.

To identify genetic variants that significantly influence ACD, and to determine if such genes (if any) affects PACG risk, we conducted a two-staged study, first a GWAS on a total of 4,484 population-based individuals of Indian and Malay ethnicity from Singapore, and Chinese from Beijing, China. Secondly, the identified QTLs for ACD were assessed for association in PACG case cohorts.

## Results

### Identification of a QTL for ACD

After sample and genotyping QC, a total of 1752 (Singapore Malay Eye Study, SiMES), 1860 (Singapore Indian Eye Study, SINDI), and 872 (Beijing Eye Study, BES) individuals with complete data for ACD measurements, age and gender were available for GWA analysis. We measured the association between ACD and individual SNP genotypes using linear regression, modeling for a trend-per-copy effect on the minor allele. Additional adjustments were made for age, gender, and the significant axes of genetic stratification. We noted a significant excess of small *P*-values at the extreme tail of the quantile-quantile distribution (**Figure S1**) accompanied by a background of minimal genomic inflation, thus indicating that there could be genuine associations between SNP genotypes and ACD. Highly suggestive evidence of association (*P* = 1.92×10^−7^) was observed at a sequence variant within *ABCC5* (rs1401999) on Chromosome 3 (**Figure S2**). We were able to replicate of this observation in a further 824 population-based samples of Chinese descent from Beijing, China (*P* = 0.011) using Sanger sequencing, leading to genome-wide significant association with ACD upon meta-analysis of all 5,308 population-based samples (β = −0.045 mm ACD per-copy of the minor allele (C allele), *P* = 8.17×10^−9^; **Table 1**). Furthermore, attesting to the robustness of our findings, we observed a similar magnitude of association when using left eye ACD measurements of SiMES and SINDI cohorts (β = −0.051, P = 8.15×10^−4^ and β = −0.045, P = 8.11×10^−5^ respectively), where left eye ACD data were also available.

**Table 1:** Quantitative trait analysis between *ABCC5* rs1401999 and anterior chamber depth in SIMES, SINDI, and BES.

Collection	N	Minor Allele	β	SE	*P*gc	MAF
SIMES	1752	C	−0.056	0.0149	1.76×10^−4^	0.15
SINDI	1860	C	−0.041	0.0115	3.97×10^−4^	0.41
BES1	872	C	−0.026	0.0196	0.19	0.16
BES2	824	C	−0.058	0.0227	0.011	0.16
All BES	1696	C	−0.040	0.0148	0.0075	0.16
Meta-analysis*	5308	C	−0.045	0.00775	8.17×10^−9^	

SIMES: Singapore Malay Eye Study (typed with Illumina 610K GWAS chip).

SINDI: Singapore Indian Eye Study (typed with Illumina 610K GWAS chip).

BES1: Beijing Eye Study typed with Illumina 610K GWAS chip.

BES2: Beijing Eye Study typed with direct sequencing.

β: Per-allele effect size of *ABCC5* rs1401999 on anterior chamber depth.

SE: Standard error for β.

*P*gc: Genomic control corrected *P*-value.

MAF: Minor allele frequency.

*: I^2^-index for heterogeneity = 0%.

Additionally, as both ACD and axial length are distance measurements on the axial direction of the eye globe, previously described to share genetic factors to a certain degree [15], we also assessed the effect of *ABCC5* rs1401999 on axial length. We found the association between rs1401999 and axial length to be much weaker compared to that observed with ACD (*P*-meta = 0.000615, **Table S1**).

### Association between *ABCC5* rs1401999 and PACG

**Figure 1:**
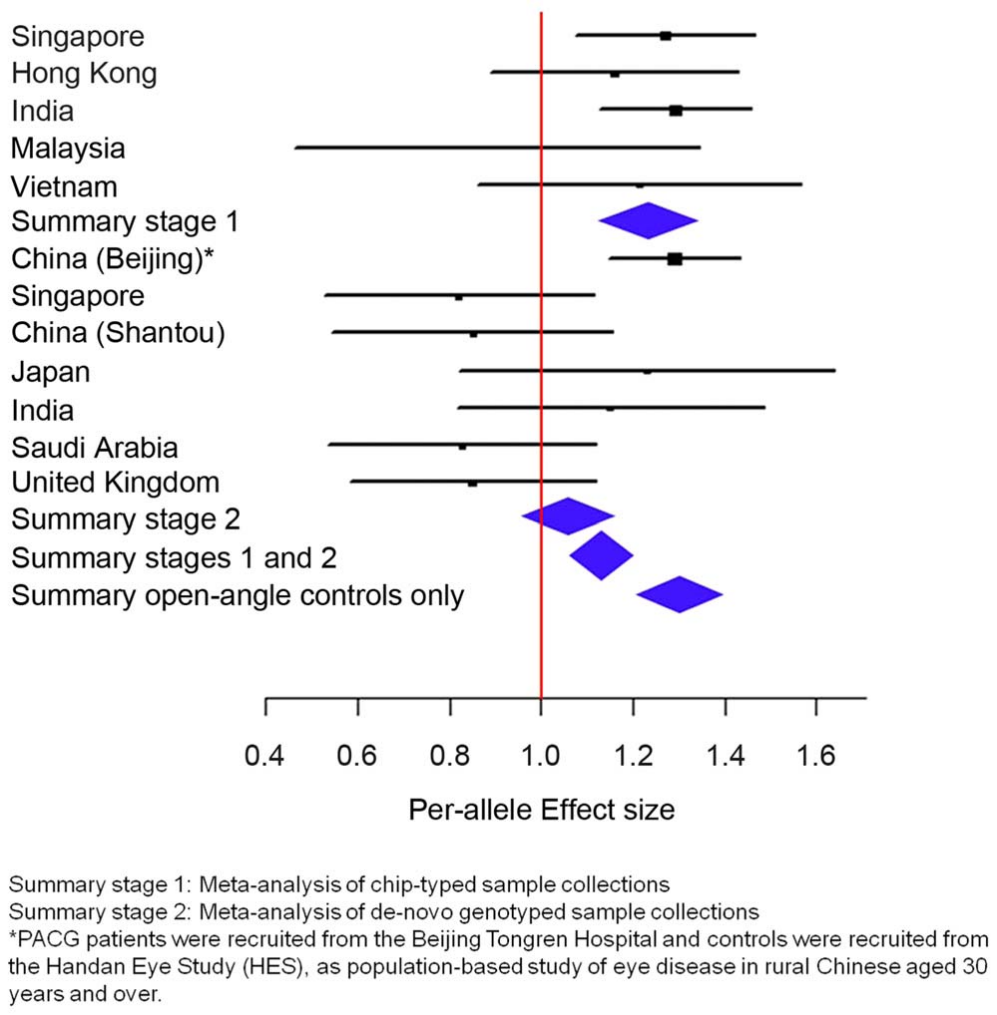
Association analysis between *ABCC5* rs1401999 and susceptibility to primary angle closure glaucoma (PACG). The PACG sample collections have been described elsewhere [6]. The vertical line represents a per-allele odds ratio of 1.00. The oblongs represent point estimates (referring to the per-allele odds ratio), with the height of the oblongs inversely proportional to the standard error of the point estimates. Horizontal lines indicate the 95% confidence interval for each point estimate. Meta-analyses of samples are reflected by blue diamonds. The width of the diamonds indicates their 95% confidence intervals. All point estimates in Stage 1 have been adjusted for the top axes of genetic stratification using logistic regression.

For the second analysis, we examined if this variant was associated with PACG, and proceeded to conduct analysis of 1,854 PACG cases and 9,608 controls from 5 cohorts (**Table S2**), all genotyped with Illumina SNP-arrays, and a further 2,422 cases and up to 9,193 controls from 7 independent collections genotyped using the Sequenom MassArray or Taqman platforms. As there was significant heterogeneity for the effect of *ABCC5* rs1401999 and PACG risk between the 12 sample collections (*P*_heterogeneity_ = 0.0047, I^2^-index = 60.6%; **Figure 1** **and** **Table 2**), we looked for sources of possible heterogeneity within the sample collections [16], the most obvious of which are the use of clean, open angle controls in some collections (see ‘Selection of controls without angle closure’ in methods), and un-ascertained population-based controls in others. This could be important in the context of this study as the prevalence of at-risk population with inherent angle closure is as high as 10% in Asian populations [3], [17]. Overall, we noted modest evidence of association (per-allele odds ratio = 1.13, 95% confidence interval = 1.06–1.22; *P* = 0.00046) between the minor allele (C allele) of rs1401999 and PACG when all 12 case-control collections were considered, and this association was augmented when we only included pre-selected sample collections where the controls had definite open angles (N = 3,458 PACG cases and N = 3,831 controls, per-allele OR = 1.30, *P* = 7.45×10^−9^; **Figure 1** and **Table 2**).

**Table 2:** Association analysis between *ABCC5* rs1401999 and primary angle closure glaucoma in all chip-typed sample collections (top panel), de-novo genotyped sample collections (middle panel), and PACG cases and clinically certified controls with open angles (bottom panel).

Stage 1 (Chip-typed sample collections)				
Collection	MAF case	MAF control	OR	*P*
Singapore	0.135	0.109	1.27	0.017
Hong Kong	0.147	0.132	1.16	0.38
India	0.493	0.408	1.29	2.83×10^−5^
Malaysia*	0.175	0.150	1.21	0.38
Vietnam	0.143	0.121	1.21	0.25
Meta-analysis (Stage 1)			1.23	9.84×10^−5^ (I^2^ = 0.0%)

Stage 2 (De-novo genotyped sample collections)				
Collection	MAF case	MAF control	OR	*P*
China (Beijing)†	0.173	0.139	1.29	0.00045
Singapore	0.120	0.143	0.82	0.17
China (Shantou)	0.131	0.150	0.85	0.31
Japan	0.136	0.113	1.23	0.32
India	0.477	0.442	1.15	0.41
Saudi Arabia	0.432	0.484	0.83	0.16
United Kingdom	0.412	0.453	0.85	0.22
Meta-analysis (Stage 2)			1.06	0.23 (I^2^ = 67.5%)
Meta-analysis (Stages 1 and 2)			1.13	0.00046 (I^2^ = 60.6%)

*ABCC5* rs1401999 genotyped in PACG cases and clinically certified open-angled controls only				
Collection	MAF case	MAF control	OR	*P*
Singapore	0.132	0.109	1.24	0.026
Hong Kong	0.159	0.103	1.64	0.012
India	0.489	0.429	1.28	0.0047
Beijing†	0.173	0.139	1.29	0.00045
Japan	0.136	0.113	1.23	0.32
Meta-analysis			1.30	7.45×10^−9^ (I^2^ = 0.0%)

MAF case: Minor allele frequency in PACG cases.

MAF control: Minor allele frequency in controls.

OR: Odds ratio.

*P*: *P*-value for association with PACG.

I^2^: I-squared index for between-collection heterogeneity.

* Results here are presented based on raw minor allele frequency counts without further adjustment.

† PACG patients were recruited from the Beijing Tongren Hospital and controls were recruited from the Handan Eye Study (HES), a population-based study of eye disease in rural Chinese aged 30 years and over.

We also performed conditional analyses of the ABCC5 variant with the previously implicated PACG loci [6] and observed no change in either the odds ratio or p-values of the association. (Table S3).

### Expression of *ABCC5* in eye tissues

RT-PCR analysis on human ocular tissues demonstrated that *ABCC5* is expressed in anterior segment structures relevant to PACG such as the iris, ciliary body, and lens (**Figure S3**). Additionally, *Abcc5* message and protein were confirmed in mouse ocular tissues using *in situ* hybridization and immunohistochemistry (**Figure S4, S5**).

## Discussion

In this study, a GWAS of ACD in Singaporean Indians and Malays and Chinese from Beijing China found rs1401999 within *ABCC5* to contribute to the normal variation of ACD, a quantitative trait relevant to PACG. Interestingly, none of the previously identified PACG-associated genetic variants [6] were significantly associated with ACD [18]. We also demonstrated the association of rs1401999 with PACG using case-control cohorts from multiple populations across Asia. Importantly this association surpassed GWAS significance when the analysis was confined to control pre-selected to have open angles.

*ABCC5*, also known as multidrug resistance protein 5 (MRP5), has been shown to participate in tissue defense and cellular signal transduction through efflux of anticancer drugs, toxicants and a second messenger cGMP [19]. [20], [21]. It is expressed in most human tissues, including the cornea [22], retinal pigment epithelium and retina of the eye [23]. We also noted *ABCC5* expression in ocular structures relevant to PACG such as the iris, ciliary body, and lens. However, its exact role in the context of PACG is not yet known. The significant association between *ABCC5* rs1401999 with a shallower ACD argues favorably for a role in eye growth, particularly that of the anterior segment. Intriguingly, a study in zebrafish suggested that Abcc5 may play an active role in eye development through the regulation of intracellular cGMP levels. Zebrafish Abcc5, which shares 73% amino acid sequence identity with human ABCC5, is highly expressed in the lens of the developing eye [24]. Notably, the blockage of endogenous ABCC5 activity by its dominant-negative was shown to retard development, producing smaller eyes as well as overall reduction of body length and pigmentation of embryos [24]. *Abcc5* knockout mice have been generated but an evaluation of their eyes was not reported [25]. A developmental role for ABCC5 in mammalian eyes therefore remains to be defined and will require further detailed studies in model organisms.

In addition, the linkage disequilibrium (LD) block that includes rs1401999 and *ABCC5*, also includes the presenilin-associated rhomboid-like (*PARL*) gene, 5-hydroxytryptamine receptor 3D (*HTR3D*) gene and the 5-hydroxytryptamine receptor 3C (*HTR3C*) gene (**Figure S6**). It is thus possible that rs1401999 might simply be in LD with an as yet unidentified causal variant, and it remains unclear whether the causal alleles or group of alleles influence *ABCC5* or any of the neighboring genes to influence ACD. However, the clear expression within ocular tissues and a possible role in eye development make *ABCC5* a rather attractive candidate gene for ACD. Indeed, re-sequencing of the region will be necessary to identify novel potentially functional polymorphisms related to PACG pathogenesis.

The prevalence of the pre-cursor stage of PACG, namely narrow angles, is about 10% in many Asian populations [3], [17]. Given the association between angle closure and shallow ACD [11] it is therefore appropriate to remove the ‘at-risk individuals’ from the control population in order to assess the true relationship between ACD QTLs and PACG. Unsurprisingly, the evidence of association between rs1401999 and PACG was augmented when we only included sample collections where the controls had definite open angles. Similar observations have also been seen with very recent studies on Alzheimer's disease, where the inclusion of general population-based controls resulted in significant underestimation of the odds ratio conferred by the disease-associated SNP compared to when properly matched, risk-free controls were applied, [26]. [27], [28]. We caution that the modest statistical evidence reported here for rs1401999, when examined in a total of 4,276 PACG cases and 18,801 controls, is at least 5 orders of magnitude below that of the three PACG-associated variants from our recent GWAS study [6]. Clinical studies have shown that ACD is only a modest determinant of angle width [29]. Therefore, this may explain why an ACD controlling gene such as ABCC5 would only confer a relatively small effect on PACG disease itself. Incidentally, the proportion of PACG risk explained by ABCC5 rs1401999 is 0.35% (95% confidence interval = 0.01 to 1.2%). Our study highlights the fact that even larger sample sizes may be necessary to dissect and conclusively identify the possible modifiers of genetic risk conferred by variants of modest effects, particularly when they exert their action on disease pathogenesis via endophenotypes. The following observations can also be drawn from this study. It is important that all sample collections are included and assessed transparently especially when drawn from diverse populations. Secondly, despite the broad-based success in the use of large numbers of unselected, population-based controls in genetic studies [30], [31], [32], [33], [34], [35] the deployment of controls with proper clinical phenotyping and documentation will often assist in more definitive identification of susceptibility genes. A comprehensive examination of all variation around *ABCC5* using targeted deep re-sequencing is now necessary to parse the true association signal in an effort to more completely understand the role of this gene in ACD and PACG.

In summary our study identified a common genetic variant within *ABCC5* as being significantly associated with ACD, which was also associated with a modest risk of PACG. Our findings are largely in keeping with the anatomical risk factors of individual susceptibility to PACG in the eye, whereby shallower ACD is a cardinal clinical and pathogenic feature, predisposing the eye to more ‘crowded’ anterior segment and thus increasing the risk of PACG. Our study provides further clues to genetic mechanisms underlying this major global cause of blindness.

## Materials and Methods

### Sample collections analyzed for Anterior Chamber Depth

Anterior chamber depth (ACD) measurements were derived from three population based samples: the Singapore Malay Eye Study (SiMES), the Singapore Indian Eye Study (SINDI) and the Beijing Eye Study (BES).

#### SiMES

The Singapore Malay Eye Study (SiMES) was a population-based, cross-sectional study of 3280 Malay adults aged 40 to 79 years. Details of the SiMES design, sampling plan, and methods have been reported elsewhere [36]. In brief, an age-stratified random sampling of all Malay adults, aged 40 to 80 years, residing in 15 residential districts in the south- western part of Singapore was drawn from the computer-generated random list of 16,069 Malay names provided by the Ministry of Home Affairs. A total of 1400 names from each decade of age (40–49, 50–59, 60–69, and 70–79 years), or 5600 names, were selected. Of these, 4168 individuals (74.4%) were determined to be eligible to participate. A person was considered ineligible if he or she had moved from the residential address, had not lived there in the past 6 months, was deceased, or was terminally ill. Of the 4168 eligible individuals, 3280 participants (78.7%) took part in the study. The study was conducted from August, 2004 to June, 2006.

##### SINDI

As with SiMES, the Singapore Indian Eye Study (SINDI) was a population-based, cross-sectional epidemiological study, but of ethnic Indian adults aged between 40 and 80+ years residing in Singapore. The Ministry of Home Affairs provided an initial computer-generated list of Indian names derived from a simple random sampling of all ethnic Indian adults aged 40–80+ years of age residing in 15 residential districts in south-western Singapore. From this list, a final sampling frame of 6,350 ethnic Indian residents was derived using an age-stratified random sampling strategy similar to SiMES. SINDI was conducted from March, 2007 to December, 2009 and recruited 3,400 (75% response rate) participants [37].

##### BES

The Beijing Eye Study was a population-based, cross-sectional study of Chinese adults aged 40+ years and residing in 4 communities in the urban district of Haidian in the North of Central Beijing and in 3 communities in the village area of Yufa of the Daxing District south of Beijing [38]. At the time of the first survey in the year 2001, the 7 communities had a total population of 5324 individuals aged 40 years or older and eligible to take part in the study. In total, 4439 individuals participated in the eye examination (83.4% response rate). In the year 2006, when blood samples were taken, the study was repeated by re-inviting all participants from the survey from 2001 to be re-examined with 3251 subjects participating (73.3% response rate).

##### Measurement and analysis of Anterior Chamber Depth (ACD)

ACD was measured using the IOLMaster (Carl Zeiss Meditec, Dublin, CA). Five readings were obtained and the average computed. The signal-to-noise ratio for all readings were >2.0, which indicate that a clear signal was obtained when performing the measurement. All the readings were within 0.05 mm of the one with the highest signal-to-noise ratio.

The ACD measurements used in the GWAS excluded any measurements from any eye which were pseudophakic or aphakic. For collections with data from two phakic eyes (SiMES, SINDI), individuals were excluded whose ACD measurements between the two eyes differed more than 0.2 mm (which represented the top ∼20th percentile of symmetrical data) which gave a good correlation between the left and the right eye (r^2^>0.95 in both SiMES and SINDI). However, given that BES only had measurements for the right eye; final meta-analysis used ACD measurements taken from the right eye in all three cohorts.

##### PACG case-control cohorts

The subjects for the PACG case-control study were compiled from 11 independent sample collections enrolled from 8 different countries; and have been described previously [6]. Furthermore, we have included an additional PACG case-control collection from Japan (136 cases and 419 controls) as well as an additional 436 PACG cases from the Beijing site. The PACG cases and controls were defined using the same criteria as described previously [6].

###### Selection of controls without angle closure

These controls were selected from within the population-based samples based on robust clinical criteria. A control was defined as having an intraocular pressure (IOP)<21 mmHg with open angles (on gonioscopy) in all quadrants, healthy optic nerves and normal visual fields, and no previous intraocular surgery.

##### Ethics

All involved studies were conducted in accordance with the principles of the Declaration of Helsinki. Study procedures and protocols were approved by the Institutional Review Board of each local institution involved in the study, and all study participants provided written informed consent at the recruitment into the studies.

##### Genotyping

###### ACD GWAS

Genotyping in the following sample collections (SIMES, N = 1752; SINDI, N = 1860; BES1, N = 872) was performed using the Illumina 610K Quad BeadChips following manufacturer instructions after genomic DNA were extracted from participants using standard laboratory techniques. Genotyping of SNP ABCC5 rs1401999 in an additional 824 participants from the Beijing Eye Study (termed BES2) was performed using direct capillary sequencing.

###### PACG sample collections

Genome-wide genotyping was performed for a total of 1,854 PACG cases and 9,608 controls using Illumina SNP-arrays. A further 1,917 PACG cases and up to 8,943 controls were genotyped using the Sequenom MassArray and Taqman real-time PCR method (**Table S2**).

##### Statistical analysis

Genome-wide per-cohort and meta-analysis of ACD for all three sample collections was performed using standard procedures as previously described [39], [40], [41]. A selection of stringent QC filters were applied to remove poorly performing SNPs and samples using tools implemented in PLINK version 1.7 [42]. The QC criteria were as follows: SNPs that had >5% of missing genotypes, gross departure from Hardy-Weinberg equilibrium (test for HWE showing *P*<10^−6^) or were of minor allele frequency below 1% were excluded from downstream analysis. For sample QC, samples with an overall genotyping call rate of <95% were excluded from analysis. Principal component (PC) analysis was undertaken to account for spurious associations resulting from ancestral differences of individual SNPs. PC plots were performed using the R statistical program package (www.r-project.org/).

For the GWAS on ACD, linear regression was performed to test for association between SNP genotypes and ACD as implemented by PLINK (version 1.06). Individual SNP genotypes were coded according to the number of copies of the minor allele present: 0 for the wild-type genotype, 1 for heterozygotes, and 2 for homozygote variants. A trend test using linear regression was used for primary association testing between genotypes and ACD as a quantitative trait, adjusting for age, gender, and the significant axes of genetic stratification. Meta-analysis across SiMES, SINDI and BES was performed using the inverse-variance, fixed effects model in order to obtain a combined point estimate of the overall effect size (β) coefficients and its corresponding standard error (SE). Inter-cohort heterogeneity was assessed with the Cochran's Q statistic and its accompanying I^2^ index. Quantile-quantile (QQ) and Manhattan plots were created using the software R (www.r-project.org). After sample and genotyping QC, a total of 1752, 1860, 872 individuals with complete data for ACD measurements, age and gender were available for SiMES, SINDI and BES individual GWAS. The overall genomic inflation factor for the meta-analysis of the three sample collections was minimal (λgc = 1.036; see **Figure S1**). We considered *P*<5×10^−8^ as genome-wide significant, and the previously used threshold for genome-wide significance (*P*<5×10^−7^) as ‘highly suggestive evidence of association [43].

Descriptions of the GWAS datasets used in the current study, principal component analysis, and adjustment for population stratification have been described elsewhere (**Table S4**) [39], [40], [41].

For the PACG sample collections, the analysis was performed as previously described, with associations between *ABCC5* rs1401999 and PACG modeled using logistic regression.

##### Power calculations

A power calculation was conducted for bringing forward genome-wide significant SNPs from the ACD quantitative trait analysis to the PACG case control analysis (for 4,276 cases and 18,801 controls) (Supplementary Table S5). The power calculation is consistent with the findings we report in the current manuscript.

##### Expression analysis

###### RT-PCR in human ocular tissues

Expression of *ABCC5* was assessed by semi quantitative reverse transcription PCR (RT-PCR) using *ABCC5* specific primers (forward 5′- ATTGGCATTGTGGGGCGGAC -3′ and reverse 5′- CCTCTCCAGGGCATCCCAAATC -3′) on total RNA extracted from a variety of ocular tissues (anterior sclera, cornea, iris, ciliary body, trabecular meshwork, lens, lens capsule, retina and retinal pigment epithelium, optic nerve head and optic nerve) with TRIzol Reagent (Invitrogen, Carlsbad, California) in accordance with the manufacturer's protocol. First-strand cDNA synthesis was performed with SuperScript First-Strand Synthesis System for RT-PCR (Invitrogen, Carlsbad, California). Semi quantitative RT-PCR was performed according to manufacturer's protocol, with the SYBR Green Master Mix (Invitrogen, Carlsbad, California) using the above *ABCC5* primers. The resulting PCR products were separated on a 2% agarose gel and visualized by ethidium bromide staining. The ubiquitously expressed beta-actin *(ACTB)* gene was amplified using specific primers (forward 5′- CCAACCGCGAGAAGATGA -3′ and reverse 5′- CCAGAGGCGTACAGGGATAG-3′) and used as amplification and normalizing control. The *ABCC5* primer sequences were derived from NCBI Reference Sequence: NM_005688.2.

###### *In situ* hybridization

*In situ* hybridization was performed on 12-µm-thick 4% paraformaldehyde (PFA) fixed ocular sections using Dig-labeled riboprobe. Digoxigenin-labeled (DIG-labeled) riboprobes for mouse *Abcc5* were transcribed from cDNA clones (Open Biosystems clone ID: 6839816). For anti-sense probe generation, the plasmid was digested with EcoRI and transcribed with T3 polymerase. While for the sense probe Not1 and T7 were used. For this procedure, C57BL/6J or A/J mice were perfused transcardially with 4% PFA in Phosphate buffered saline (PBS). Eyes were postfixed in 4% PFA overnight, cryoprotected in 30% sucrose, and embedded in Optimal Cutting Temperature embedding medium (Tissue-Tek O.C.T. Compound, Sakura Finetek U.S.A., Inc., Torrance, CA). Frozen sections were air dried (10 min), postfixed (4% PFA; 10 min) and acetylated with 0.25% acetic anhydride in 0.1 M triethanolamine (TEA). Intercalated washes were done with PBS and after the last wash the sections were incubated overnight at 65°C with hybridization solution [50% formamide, 1× Hybe solution (Sigma-Aldrich, St. Louis, MO), 1 mg/ml yeast RNA] containing 1 µg/ml Dig-labeled riboprobes. After hybridization, slides were washed with 0.2× SSC at 72°C for 1 h, and endogenous peroxidases were quenched with a solution of 0.1% sodium azide and for 10 min. Bound probes were detected with an POD-conjugated anti-Dig antibody. The detection of hybridized mRNA in sections was performed using the Cy-3 Tyramide Signal Amplification System (PerkinElmer, Waltham, MA).

###### Immunohistochemistry

Enucleated eyes from A/J mice were embedded in Optimal Cutting Temperature embedding medium (Tissue-Tek O.C.T. Compound, Sakura Finetek U.S.A., Inc., Torrance, CA). The eyes were cryo-sectioned (14 µm) and transferred to glass slides. Cryosections were air dried for 10 min at room temperature, fixed for 10 min in 4% paraformaldehyde, followed by two washes (5 min each) in phosphate buffered saline (PBS). Sections were blocked 30 min at room temperature with 10% normal donkey serum. Primary antibodies were applied for 1 hr at room temperature using polyclonal goat anti-ABCC5 antibody (diluted 1∶300; Catalog No. sc-5781, Santacruz Biotechnology Inc., Santacruz, CA). Primary antibody was removed by three washes (5 min each) in PBS and the sections were treated for 1 hr at room temperature with AlexaFluor conjugated secondary antibodies (1∶200 dilution, Jackson-ImmunoResearch, West Grove, PA) diluted in 1% normal donkey serum and 10 mg/mL BSA in PBS. After three washes in PBS, the sections were mounted (Fluormount, Sigma-Aldrich, St. Louis, MO) and viewed by fluorescence microscopy. All photomicrographs were taken with identical camera settings. For peptide blocking experiments, the antibody was preincubated with 10× concentration of the blocking peptide (sc-5781P, Santacruz Biotechnology Inc., Santacruz, CA), incubated for 2 h at room temperature prior to treating it with the sections.

#### Supporting Information

Figure S1 Quantile-quantile plot of *P*-values from the meta-analysis of ACD across the three independent sample collections with genome-wide genotyping data (Singapore Malays, N = 1752; Singapore Indians, N = 1860; and Chinese from Beijing, N = 872) totaling 4,484 individuals.
(DOC)

Figure S2 Manhattan plot for genome-wide meta-analysis of anterior chamber depth in three populations with genome-wide genotyping data (Singapore Malays, N = 1752; Singapore Indians, N = 1860; and Chinese from Beijing, N = 872) totaling 4,484 individuals. A further 824 Chinese samples from Beijing which were genotyped via direct sequencing for rs1401999 are not included in this Manhattan plot.
(DOC)

Figure S3 Expression analysis of *ABCC5* in human ocular tissues: The *ABCC5* specific 249 bp RT-PCR product was observed for anterior sclera (AS), cornea (cornea epithelium, CE; corneal stroma, CS and cornea endothelium, CEn), iris (I), trabecular meshwork (TM), ciliary body (CB), lens (L), lens capsule (LC), retina and retinal pigment epithelium (R), optic nerve head (ONH) and optic nerve (ON). The ubiquitously expressed gene, *ACTB* was used as the normalizing control. A no template sample acted as the negative control (NC) to ensure non-contamination of the RT-PCR reaction mix. M denotes molecular-weight marker.
(DOC)

Figure S4 *Abcc5* is expressed in multiple ocular tissues that may participate in the pathogenesis of PACG. RNA *In situ* hybridization with an antisense probe (AS) shows that *Abcc5* mRNA is expressed in: A) iris (I), B) ciliary body (CB), C) cornea, and D) in the outer nuclear layer (ONL) inner nuclear layer (INL) and ganglion cell layer (GCL) of the retina. The middle panel shows a merged image of AS staining and DAPI. *In situ hybridization* with asense probe (S) control is shown in the right panel. Scale bar, 50 µm.
(DOC)

Figure S5 Immunohistochemical localization of ABCC5 in ocular tissues: Cryosections of whole eyes from wild-type A/J mice were imaged using fluorescence microscopy. ABCC5 is present in: A) iris (I), cornea (C), B) ciliary body (CB), and C) retina, in the inner segment (IS), inner nuclear layer (INL) and Muller cell processes in the inner plexifom layer (IPL). The right panel shows images with immunostaining blocked by a competing ABCC5 peptide(+ Pep).Scale bar, 50 µm.
(DOC)

Figure S6 Regional linkage disequilibrium (LD) plot for *ABCC5* and its flanking region (Chr. 3). This is plotted using the D' algorithm.*ABCC5* rs1401999 is labeled with an arrow. D' = 1 represents complete LD.
(DOC)

Table S1 Quantitative trait analysis between *ABCC5* rs1401999 and axial length.
(DOC)

Table S2 PACG samples collections and genotyping methodology for *ABCC5* rs1401999.
(DOC)

Table S3 Association analysis between ABCC5 rs1401999 and PACG in the GWAS collections. Additional adjustments compensating for the allelic dosages at PLEKHA7 rs11024102, COL11A1 rs3753841, and rs1015213 are also performed in addition.
(DOC)

Table S4 Baseline characteristics of sample collections used in quantitative trait analysis for anterior chamber depth (genotyped individuals passing quality checks from SiMES, SINDI and BES).
(DOC)

Table S5 Study power as a function of minor allele frequency and per-allele odds ratios. Cells in yellow highlights fulfill >80% statistical power to achieve P = 1×10^−4^.
(DOC)
