# Correction: ABCC5, a Gene That Influences the Anterior Chamber Depth, Is Associated with Primary Angle Closure Glaucoma

**DOI:** 10.1371/journal.pgen.1004352

**Published:** 2014-04-04

**Authors:** The PLOS Genetics Staff

Dr. Vernon K Y Yong is not included in the author byline. He should be listed as the forty second author and affiliated with Department of Ophthalmology, Tan Tock Seng Hospital, Singapore.
