# Supplementary figures and images for: ABCC5, a Gene That Influences the Anterior Chamber Depth, Is Associated with Primary Angle Closure Glaucoma
Source: PLoS Genet. 2014 Mar 6;10(3):e1004089. doi: 10.1371/journal.pgen.1004089 (PMC3945113; doi:10.1371/journal.pgen.1004089)

Figure S1


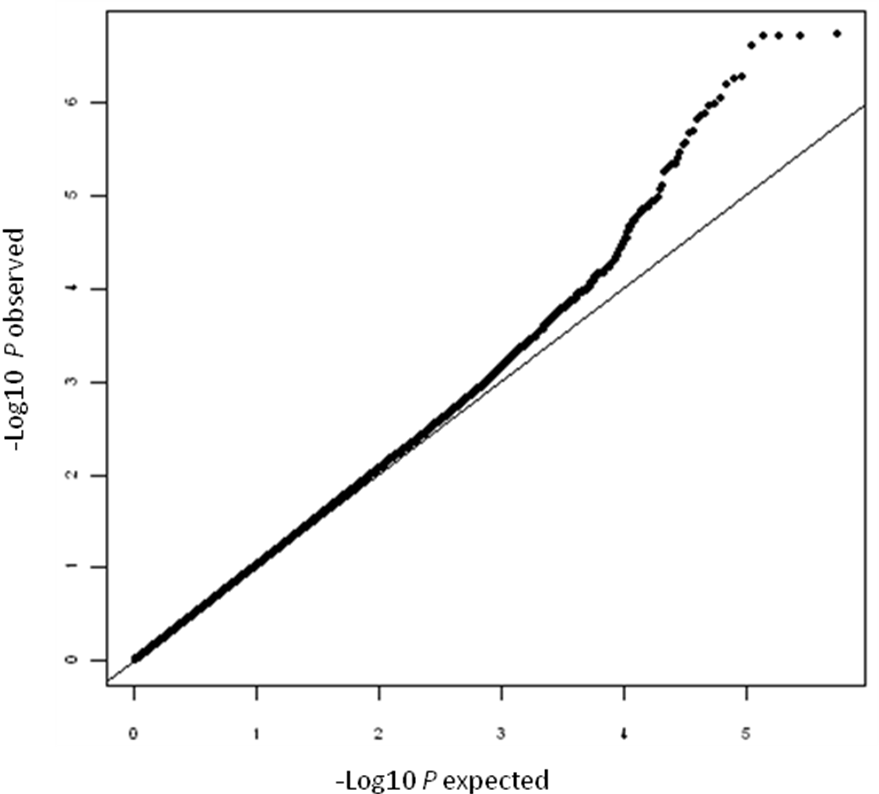

Supplement: Figure S1 — Quantile-quantile plot of P-values from the meta-analysis of ACD across the three independent sample collections with genome-wide genotyping data (Singapore Malays, N = 1752; Singapore Indians, N = 1860; and Chinese from Beijing, N = 872) totaling 4,484 individuals. (DOC) [file pgen.1004089.s001.doc]

Figure S3


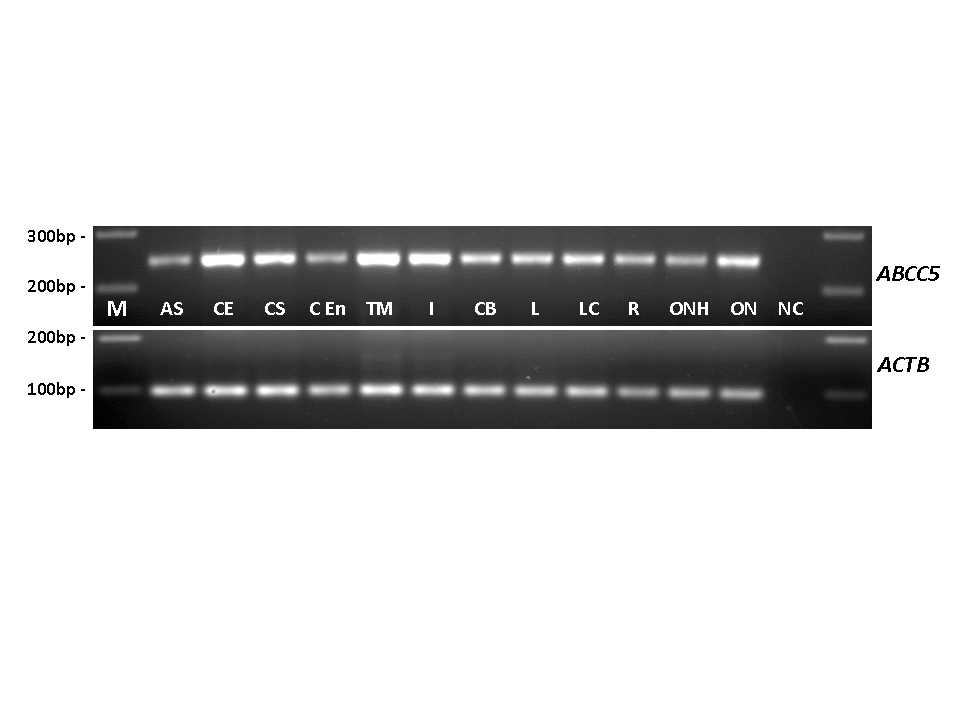

Supplement: Figure S3 — Expression analysis of ABCC5 in human ocular tissues: The ABCC5 specific 249 bp RT-PCR product was observed for anterior sclera (AS), cornea (cornea epithelium, CE; corneal stroma, CS and cornea endothelium, CEn), iris (I), trabecular meshwork (TM), ciliary body (CB), lens (L), lens capsule (LC), retina and retinal pigment epithelium (R), optic nerve head (ONH) and optic nerve (ON). The ubiquitously expressed gene, ACTB was used as the normalizing control. A no template sample acted as the negative control (NC) to ensure non-contamination of the RT-PCR reaction mix. M denotes molecular-weight marker. (DOC) [file pgen.1004089.s003.doc]

Figure S4


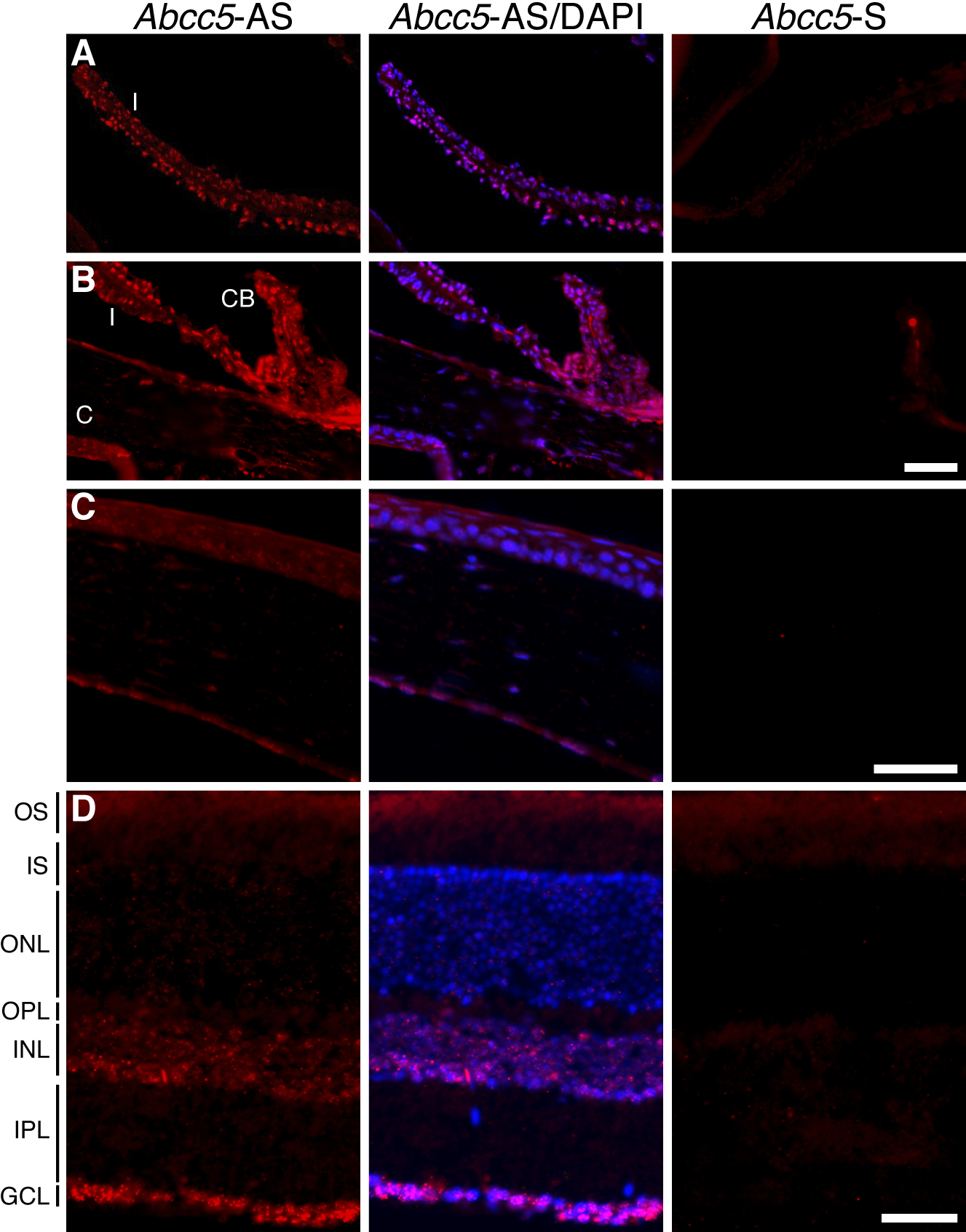

Supplement: Figure S4 — Abcc5 is expressed in multiple ocular tissues that may participate in the pathogenesis of PACG. RNA In situ hybridization with an antisense probe (AS) shows that Abcc5 mRNA is expressed in: A) iris (I), B) ciliary body (CB), C) cornea, and D) in the outer nuclear layer (ONL) inner nuclear layer (INL) and ganglion cell layer (GCL) of the retina. The middle panel shows a merged image of AS staining and DAPI. In situ hybridization with asense probe (S) control is shown in the right panel. Scale bar, 50 µm. (DOC) [file pgen.1004089.s004.doc]

Figure S5.

**
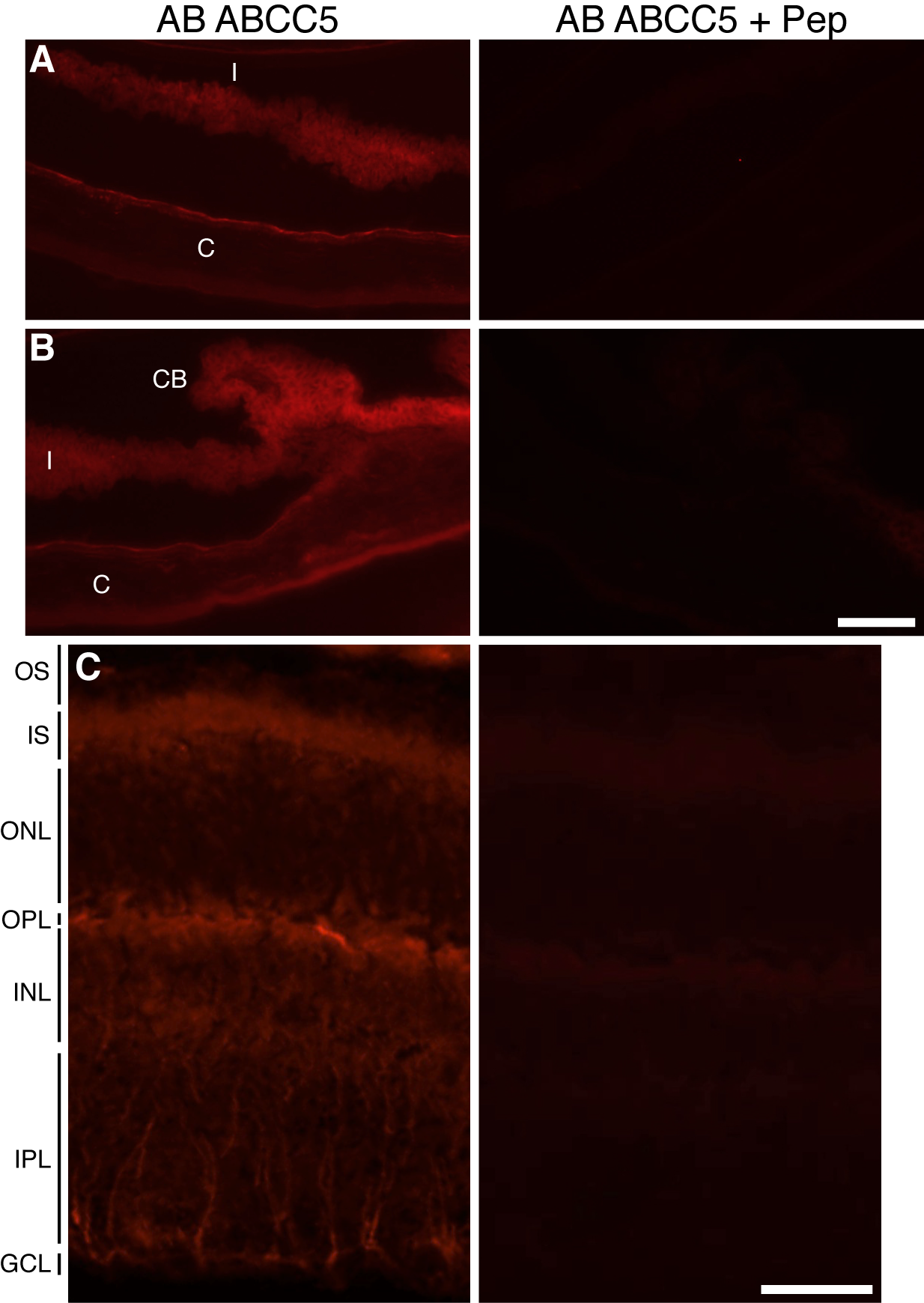
**

Supplement: Figure S5 — Immunohistochemical localization of ABCC5 in ocular tissues: Cryosections of whole eyes from wild-type A/J mice were imaged using fluorescence microscopy. ABCC5 is present in: A) iris (I), cornea (C), B) ciliary body (CB), and C) retina, in the inner segment (IS), inner nuclear layer (INL) and Muller cell processes in the inner plexifom layer (IPL). The right panel shows images with immunostaining blocked by a competing ABCC5 peptide(+ Pep).Scale bar, 50 µm. (DOC) [file pgen.1004089.s005.doc]

Figure S6

**
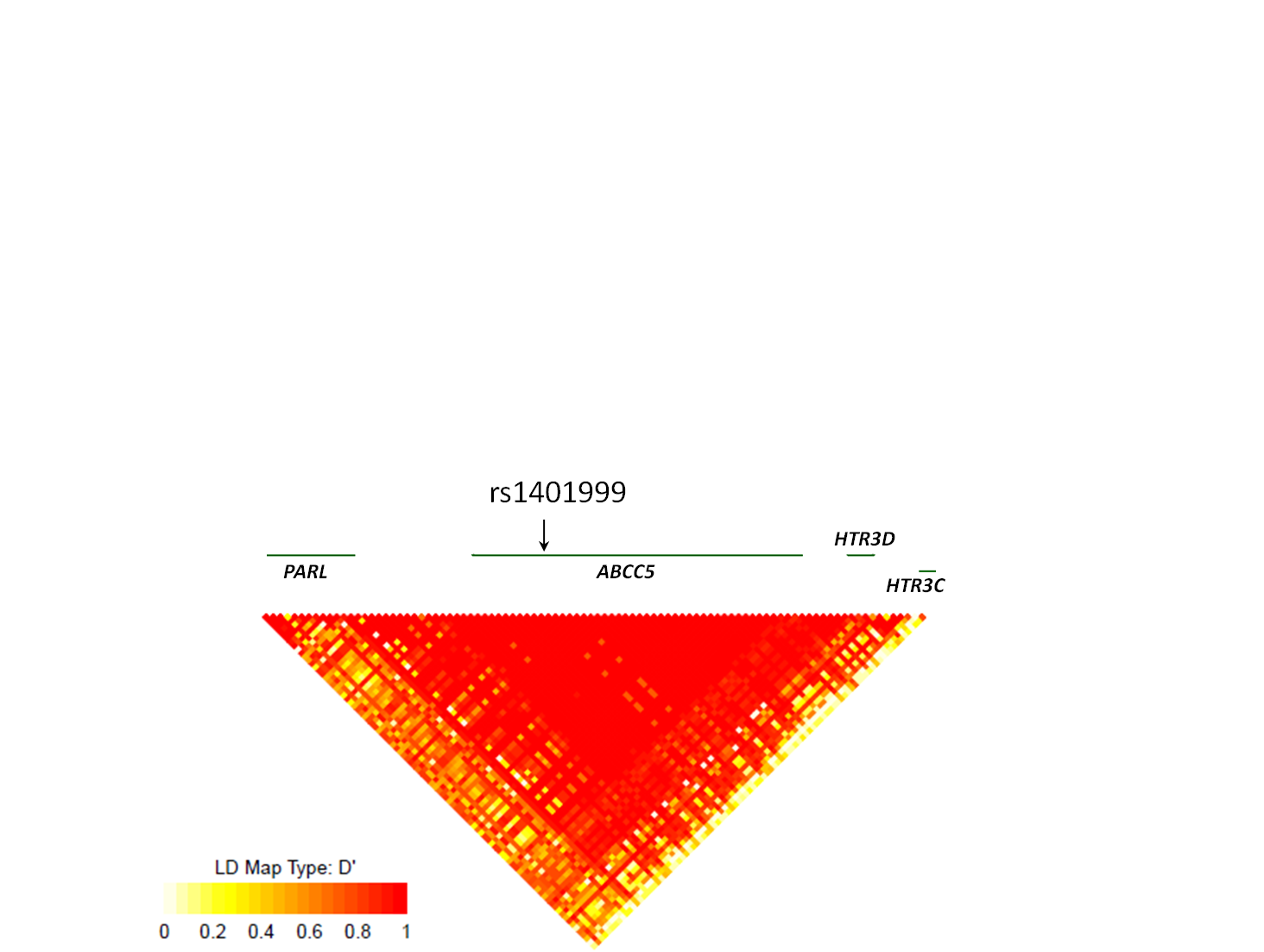
**

Supplement: Figure S6 — Regional linkage disequilibrium (LD) plot for ABCC5 and its flanking region (Chr. 3). This is plotted using the D' algorithm.ABCC5 rs1401999 is labeled with an arrow. D' = 1 represents complete LD. (DOC) [file pgen.1004089.s006.doc]
