# Supplementary material for: ABCC5, a Gene That Influences the Anterior Chamber Depth, Is Associated with Primary Angle Closure Glaucoma
Source: PLoS Genet. 2014 Mar 6;10(3):e1004089. doi: 10.1371/journal.pgen.1004089 (PMC3945113; doi:10.1371/journal.pgen.1004089)
Supplement: Table S1 — Quantitative trait analysis between ABCC5 rs1401999 and axial length. (DOC) [file pgen.1004089.s007.doc]

Table S1

Quantitative trait analysis between *ABCC5* rs1401999 and axial length.

| **Collection** | **N** | **Minor Allele** | **β** | **SE** | ***P*gc** | **MAF** |
| --- | --- | --- | --- | --- | --- | --- |
| SiMES | 2136 | C | -0.02783 | 0.04205 | 0.51 | 0.15 |
| SINDI | 2089 | C | -0.1158 | 0.03139 | 0.000231 | 0.41 |
| BES | 634 | C | -0.0662 | 0.09079 | 0.41 | 0.16 |
| Meta-analysis | 4859 | C | -0.08303 | 0.02424 | 0.000615^*^ |  |
| SIMES: Singapore Malay Eye Study  SINDI: Singapore Indian Eye Study  BES: Beijing Eye Study  N: Number of individuals with complete genotyping and axial length data.  β: Per-allele effect size of *ABCC5* rs1401999 on axial length (in mm)  SE: Standard error for β  *P*gc: Genomic control corrected *P*-value  MAF: Minor allele frequency  **P*_heterogeneity_= 0.24, I^2^ = 29.8% | | | | | | |
