# Supplementary material for: ABCC5, a Gene That Influences the Anterior Chamber Depth, Is Associated with Primary Angle Closure Glaucoma
Source: PLoS Genet. 2014 Mar 6;10(3):e1004089. doi: 10.1371/journal.pgen.1004089 (PMC3945113; doi:10.1371/journal.pgen.1004089)
Supplement: Table S2 — PACG samples collections and genotyping methodology for ABCC5 rs1401999. (DOC) [file pgen.1004089.s008.doc]

Table S2

PACG samples collections and genotyping methodology for *ABCC5* rs1401999

* denotes population-based controls.

^a^Clinically certified open-angled controls are not accompanied by *

| **Chip-genotyped cases** | | | | |
| --- | --- | --- | --- | --- |
| **Collection** | **PACG cases** | **Genotyping method** | **Controls**^a^ | **Genotyping method** |
|  | **(N)** |  | **(N)** |  |
| Singapore (Chinese) | 984 | Illumina 610K | 943 | Illumina 610K |
| Hong Kong (Chinese) | 297 | Illumina 610K | 1,044* | Illumina 610K |
| Malaysia (Malays) | 83 | Illumina 610K | 3,065* | Illumina 610K |
| India (Indians) | 337 | Illumina 610K and 660W | 2,538* | Illumina 610K |
| Vietnam (Vietnamese) | 153 | Illumina 610K | 2,018* | Illumina 660W |
| All Stage 1 | 1,854 |  | 9,608 |  |
| **De-novo genotyped cases** | | | | |
| **Collection** | **PACG cases** | **Genotyping method** | **Controls** | **Genotyping method** |
|  | **(N)** |  | **(N)** |  |
| Singapore (Chinese) | 242 | Sequenom | 1,479* | Illumina 610K |
| Beijing (Chinese)^†^ | 1,428 | Sequenom | 1,503 | Sequenom |
| Saudi (Middle Eastern descent) | 165 | Sequenom | 175* | Sequenom |
| UK (European descent) | 127 | Sequenom | 4,703* | Illumina 1.2M |
| India (Indians) | 80 | Sequenom | 309 | Sequenom |
| Shantou (Chinese) | 244 | Taqman | 605* | Taqman |
| Japan | 136 | Taqman | 419 | Taqman |
| All Stage 2 | 2,422 |  | 9,193 |  |
| **All samples** | **4,276** |  | **18,801** |  |
| ^†^ PACG patients were recruited from the Beijing Tongren Hospital and controls were recruited from the Handan Eye Study (HES), a population-based study of eye disease in rural Chinese aged 30 years and over. | | | | |
