# Supplementary material for: ABCC5, a Gene That Influences the Anterior Chamber Depth, Is Associated with Primary Angle Closure Glaucoma
Source: PLoS Genet. 2014 Mar 6;10(3):e1004089. doi: 10.1371/journal.pgen.1004089 (PMC3945113; doi:10.1371/journal.pgen.1004089)
Supplement: Table S3 — Association analysis between ABCC5 rs1401999 and PACG in the GWAS collections. Additional adjustments compensating for the allelic dosages at PLEKHA7 rs11024102, COL11A1 rs3753841, and rs1015213 are also performed in addition. (DOC) [file pgen.1004089.s009.doc]

Table S3

Association analysis between ABCC5 rs1401999 and PACG in the GWAS collections. Additional adjustments compensating for the allelic dosages at PLEKHA7 rs11024102, COL11A1 rs3753841, and rs1015213 are also performed in addition.

| **CHR** | **SNP** | **BP** | **A1** | **OR** | ***P*** | **Collection** | **Adjustments** |
| --- | --- | --- | --- | --- | --- | --- | --- |
| 3 | rs1401999 | 185161036 | G | 1.27 | 0.01657 | Singapore | unadjusted |
| 3 | rs1401999 | 185161036 | G | 1.28 | 0.01374 | Singapore | adjusted for rs11024102 |
| 3 | rs1401999 | 185161036 | G | 1.28 | 0.01469 | Singapore | adjusted for rs3753841 |
| 3 | rs1401999 | 185161036 | G | 1.28 | 0.01269 | Singapore | adjusted for rs1015213 |
|  |  |  |  |  |  |  |  |
| 3 | rs1401999 | 185161036 | G | 1.13 | 0.3683 | Hong Kong | unadjusted |
| 3 | rs1401999 | 185161036 | G | 1.13 | 0.3812 | Hong Kong | adjusted for rs11024102 |
| 3 | rs1401999 | 185161036 | G | 1.13 | 0.3777 | Hong Kong | adjusted for rs3753841 |
| 3 | rs1401999 | 185161036 | G | 1.13 | 0.3701 | Hong Kong | adjusted for rs1015213 |
|  |  |  |  |  |  |  |  |
| 3 | rs1401999 | 185161036 | G | 0.98 | 0.9411 | Malays | unadjusted |
| 3 | rs1401999 | 185161036 | G | 0.97 | 0.8914 | Malays | adjusted for rs11024102 |
| 3 | rs1401999 | 185161036 | G | 0.99 | 0.9635 | Malays | adjusted for rs3753841 |
| 3 | rs1401999 | 185161036 | G | 0.99 | 0.9566 | Malays | adjusted for rs1015213 |
|  |  |  |  |  |  |  |  |
| 3 | rs1401999 | 185161036 | G | 1.32 | 0.001171 | Indians | unadjusted |
| 3 | rs1401999 | 185161036 | G | 1.32 | 0.000991 | Indians | adjusted for rs11024102 |
| 3 | rs1401999 | 185161036 | G | 1.31 | 0.001418 | Indians | adjusted for rs3753841 |
| 3 | rs1401999 | 185161036 | G | 1.32 | 0.001173 | Indians | adjusted for rs1015213 |
|  |  |  |  |  |  |  |  |
| 3 | rs1401999 | 185161036 | G | 1.22 | 0.2405 | Vietnam | unadjusted |
| 3 | rs1401999 | 185161036 | G | 1.21 | 0.2497 | Vietnam | adjusted for rs11024102 |
| 3 | rs1401999 | 185161036 | G | 1.23 | 0.2199 | Vietnam | adjusted for rs3753841 |
| 3 | rs1401999 | 185161036 | G | 1.21 | 0.25 | Vietnam | adjusted for rs1015213 |
|  |  |  |  |  |  |  |  |
| 3 | rs1401999 | 185161036 | G | 1.24 | 5.02E-05 | meta | unadjusted |
| 3 | rs1401999 | 185161036 | G | 1.24 | 4.44E-05 | meta | adjusted for rs11024102 |
| 3 | rs1401999 | 185161036 | G | 1.24 | 5.19E-05 | meta | adjusted for rs3753841 |
| 3 | rs1401999 | 185161036 | G | 1.25 | 4.20E-05 | meta | adjusted for rs1015213 |
