# Supplementary material for: ABCC5, a Gene That Influences the Anterior Chamber Depth, Is Associated with Primary Angle Closure Glaucoma
Source: PLoS Genet. 2014 Mar 6;10(3):e1004089. doi: 10.1371/journal.pgen.1004089 (PMC3945113; doi:10.1371/journal.pgen.1004089)
Supplement: Table S4 — Baseline characteristics of sample collections used in quantitative trait analysis for anterior chamber depth (genotyped individuals passing quality checks from SiMES, SINDI and BES). (DOC) [file pgen.1004089.s010.doc]

Table S4

Baseline characteristics of sample collections used in quantitative trait analysis for anterior chamber depth (genotyped individuals passing quality checks from SiMES, SINDI and BES).

|  | **SiMES** | **SINDI** | **BES1** | **BES2** |
| --- | --- | --- | --- | --- |
| ***N*** | 1752 | 1860 | 872 | 824 |
| **%Male (%)** | 49.43 | 50.97 | 37.16 | 40.66 |
| **Age ^1^ [yr]** | 57.72 (10.84) | 56.45 (9.12) | 58.10 (9.35) | 62.38 (9.99) |
| **ACD ^1^ [mm]** | 3.13 (0.36) | 3.13 (0.37) | 2.41 (0.32) | 2.41 (0.34) |
| ^1^ mean (standard deviation) | | | | |
