# Supplementary material for: ABCC5, a Gene That Influences the Anterior Chamber Depth, Is Associated with Primary Angle Closure Glaucoma
Source: PLoS Genet. 2014 Mar 6;10(3):e1004089. doi: 10.1371/journal.pgen.1004089 (PMC3945113; doi:10.1371/journal.pgen.1004089)
Supplement: Table S5 — Study power as a function of minor allele frequency and per-allele odds ratios. Cells in yellow highlights fulfill >80% statistical power to achieve P = 1×10^−4^. (DOC) [file pgen.1004089.s011.doc]

Table S5

Study power as a function of minor allele frequency and per-allele odds ratios. Cells in yellow highlights fulfill >80% statistical power to achieve P = 1 x 10^-4^.

|  |  |  | **OR** |  |  |  |
| --- | --- | --- | --- | --- | --- | --- |
|  |  | **1.05** | **1.1** | **1.15** | **1.2** | **1.25** |
|  | **0.1** | 0.40% | 8.00% | 42.44% | 84.20% | 98.50% |
| **MAF** | **0.15** | 0.80% | 17.30% | 68.70% | 97% | >99% |
|  | **0.2** | 1.3 | 27.40% | 83.70% | >99% | >99% |
|  | **0.25** | 1.80% | 36.60% | 91.20% | >99% | >99% |
|  | **0.3** | 2.30% | 44% | 94.80% | >99% | >99% |
